# Vehicle Detection on Occupancy Grid Maps: Comparison of Five Detectors Regarding Real-Time Performance

**DOI:** 10.3390/s23031613

**Published:** 2023-02-02

**Authors:** Nils Defauw, Marielle Malfante, Olivier Antoni, Tiana Rakotovao, Suzanne Lesecq

**Affiliations:** 1 Univ. Grenoble Alpes, CEA, List, F-38000 Grenoble, France; 2 Univ. Grenoble Alpes, CEA, Leti, F-38000 Grenoble, France

**Keywords:** autonomous vehicle, self-driving car, perception, real-time, object detection, vehicle detection, Non-Maximum Suppression, bird’s eye view, occupancy grid map, deep learning, PIXOR, YOLO

## Abstract

Occupancy grid maps are widely used as an environment model that allows the fusion of different range sensor technologies in real-time for robotics applications. In an autonomous vehicle setting, occupancy grid maps are especially useful for their ability to accurately represent the position of surrounding obstacles while being robust to discrepancies between the fused sensors through the use of occupancy probabilities representing uncertainty. In this article, we propose to evaluate the applicability of real-time vehicle detection on occupancy grid maps. State of the art detectors in sensor-specific domains such as YOLOv2/YOLOv3 for images or PIXOR for LiDAR point clouds are modified to use occupancy grid maps as input and produce oriented bounding boxes enclosing vehicles as output. The five proposed detectors are trained on the Waymo Open automotive dataset and compared regarding the quality of their detections measured in terms of Average Precision (AP) and their real-time capabilities measured in Frames Per Second (FPS). Of the five detectors presented, one inspired from the PIXOR backbone reaches the highest $AP_{0.7}$ of 0.82 and runs at 20 FPS. Comparatively, two other proposed detectors inspired from YOLOv2 achieve an almost as good, with a $AP_{0.7}$ of 0.79 while running at 91 FPS. These results validate the feasibility of real-time vehicle detection on occupancy grids.

## 1. Introduction

Occupancy grid maps are among the classical approaches used in robotic perception in the last decades for building in real-time a digital model of the environment surrounding a robot [1,2,3]. An occupancy grid map models the environment as a bird’s eye view in a 2D grid composed of a finite number of cells. The occupancy grid algorithm computes the probability that the latter is occupied by an obstacle for each cell, given the measurements from on-board range sensors.

Occupancy grid maps have been extensively used to detect both the occupied and free space around mobile robots operating in controlled indoor environments [4,5,6,7,8]. Occupied cells constitute a low level abstraction of any kind of obstacles present in the environment. Such capacity of abstraction motivates the adoption of occupancy grid maps in self-driving car applications, where environments such as urban areas can be complex, unstructured and composed of obstacles of different natures, including cars, pedestrians, cyclists, buildings and various infrastructure [9,10,11,12,13,14].

The occupancy grid map framework computes the occupancy probabilities of cells by taking into account both sensor measurements and probabilistic models of sensor noises. Multiple occupancy probabilities about the same cell, but computed from multiple and heterogeneous sensors, can be combined into a unique occupancy probability by applying a well-established Bayesian fusion formula [3]. Regardless of the type of sensors, an occupancy grid map is, from an algorithmic viewpoint, a 2D matrix storing the occupancy probabilities of each cell of the grid. An occupancy probability is a number between zero and one, where a value of one (respectively, zero) means that the cell is occupied (respectively, free). An intermediate value expresses the level of confidence about the occupancy state of a cell [3].

An object present in the environment may occupy one or multiple cells depending on its size. However, the occupancy grid algorithm is restricted to the computation of the occupancy probabilities of cells without estimating the extent or the class of objects actually occupying cells. Occupancy grid maps lack, by definition, an object level representation including the position, orientation, size and class of objects. Such an object level representation is, however, necessary to perform complex tasks such as path planning or autonomous navigation in urban areas [13].

Methods for extracting object level representation from occupancy grid maps have been proposed in the literature. However, these methods require additional input data along with occupancy probabilities. Hoermann et al. [11] introduced an object detection approach that is not only based on occupancy grid maps, but also based on an estimation of the motion of cells occupied by moving obstacles. Erkent et al. [12] proposed to use RGB images jointly with occupancy grid maps to leverage image segmentation techniques by classifying each individual pixel of the image into various classes and applying thereafter projection mechanisms in order to segment the occupancy grid map accordingly. Wirges et al. [15] proposed to perform object detection on occupancy grid maps enriched with specific LiDAR information, including intensity of points and the minimum and maximum *z* coordinates of LiDAR points falling within each cell.

In this article, we present methods for detecting objects from occupancy probabilities without requiring additional information like specific sensor-related data or intermediate motion estimation of cells. We focus particularly on the detection of vehicles from occupancy grid maps. Our methods take advantage of the matrix format of occupancy grid maps to develop convolutional neural networks for predicting oriented bounding boxes enclosing vehicles.

The five detectors proposed are the product of a backbone neural network, a training procedure and a post-processing step. All backbones are inspired by the three domain-specific object detectors: YOLOv2 [16], YOLOv3 [17] and PIXOR [18]. A first contribution encompasses various modifications proposed to the original architectures to allow the prediction of oriented bounding boxes for vehicles on occupancy grid maps. A second contribution is an architectural modification proposed for detecting more vehicles per occupancy grid map. The training of the models is performed using the Waymo Open [19] automotive dataset. Finally, an optional Non-Maximum Suppression post-processing method for removing duplicate detections is tested for some detectors.

The Average Precision (AP) and Frames Per Second (FPS) of five proposed models are compared at the end. Experimental results show that two detectors based on a modified YOLOv2 [16] backbone and one detector based on a PIXOR [18] backbone achieve a classical ${\mathrm{AP}}_{0.7}$ metric greater than 0.77. Moreover, the two detectors based on the modified YOLOv2 [16] backbone reach a frame rate of 91 FPS, consistent with real-time operation regarding sensors frame rates. These results validate the approach of using convolutional neural networks for detecting vehicles on occupancy grid maps and hint at possible real-time architectures.

The article is organized as follows. Section 2 summarizes the state of the art regarding related work on object detection. Section 3 describes the five detectors that extend the existing methods of the state of the art. Section 4 presents the dataset used in this study, the evaluation metrics and the hardware platform used to conduct the experiments. Section 5 provides detailed results and a comparative analysis of the approaches. Finally, conclusion and future work directions are given in Section 6.

## 2. Related Work

This section presents background knowledge necessary to understand and contextualize the contributed object detectors on occupancy grid maps proposed in Section 3. To the best of our knowledge, there is no solution for object detection relying on occupancy grid maps containing only occupancy probabilities. Consequently, the focus of this section is on object detectors whose inputs are data in a matrix format. These approaches can be thereafter adapted to occupancy grid maps due to the analog matrix structure of the latter.

The most common form of matrix format inputs are images, which are matrices whose cells (called pixels) contain information of brightness at different electromagnetic wavelengths. Detecting objects on images is a classical deep learning task and a large corpus of articles provides efficient convolutional architectures for this task. Object detectors on images generally produce non-oriented bounding boxes as most objects do not have a well-defined orientation.

Two meta-architectures are commonly found in the literature for object detection on images: two-stage and one-stage detectors. On one hand, two-stage detectors such as the R-CNN family [20,21,22] process the input image with two different modules: the region proposal and the classifier. The region proposal module proposes regions that are likely to contain an object in the image. Hundreds or thousands of regions can be proposed for a single image. The classifier module classifies each proposed region into the considered classes (that can include a class for empty regions) and regresses precisely the bounding box around the object. The number of proposed regions being processed by the classifier network leads to a high computational cost, requiring a powerful hardware for real-time processing.

On the other hand, one-stage detectors such as SSD [23] or the YOLO family [16,17,24,25] contain a unique neural network that produces a fixed number of bounding boxes proposals for each image. These detectors have proven useful for real-time processing as their reduced computational cost allows them to operate in real-time on less computationally performing hardware platforms [26]. Originally, one-stage detectors were less accurate than two-stage approaches. Recent developments enabled one-stage detectors to reach similar results to two-stage detectors [17].

YOLOv2 [16] and YOLOv3 [17] are two successive versions of the YOLO family of one-stage detectors. Both of these detectors subdivide the input image into regularly spaced regions. Each region of this subdivision contains exactly one bounding box proposal. This bounding box proposal comes with a confidence score, which is used to consider the bounding box as a valid detection if its confidence score is above a user-defined threshold. The detectors consist of a unique fully convolutional neural network mapping input images to output detections. YOLOv3 produces better detections than YOLOv2 at the cost of a frame rate almost being halved. Both architectures are of interest.

YOLOv4 [25] is an improved version of YOLOv3 [17] and uses various methods to improve the training procedure, such as data augmentation and hyper-parameters optimization algorithms. The feature extractor used is CSPDarknet53, which is the same Darknet53 architecture as YOLOv3’s backbone, but with Cross Stage Partial (CSP) connections [27] instead of residual connections. CSP connections reduce computations without degrading the quality of the predictions.

In addition to classical pictures taken from a camera, object detection on satellite images is of interest. These images share with occupancy grid maps the property that a given object’s size does not depend on its distance from the camera. This property comes naturally from the bird’s eye point of view of satellite images and occupancy grid maps. Object detection on satellite images is well-studied as it is an important task for domains as diverse as maritime surveillance or archaeological research. As an example, one study proposes to adapt YOLOv4-LITE [28] to perform multi-scale ship detection on Synthetic Aperture Radar (SAR) images [29].

Unlike images, some data used in certain object detection tasks are sparse and unstructured. Due to the high efficiency of object detectors on matrix format inputs, some object detectors propose to first transform the data into a matrix format and apply a convolutional object detector architecture on this transformed input.

LiDAR point clouds are a typical example of such sparse inputs for which object detection is of interest. Approaches such as [15,18,30,31,32] propose to encode point clouds into a 2D bird’s eye view grid containing LiDAR-specific features such as the mean intensity of points. The encoded point cloud is then processed through a classical 2D convolutional neural network which efficiently produces the detections. Those approaches are less demanding on computation and more adapted for real-time processing on constrained hardware.

Specifically, PIXOR [18] encodes the LiDAR point cloud into a binary bird’s eye view grid whose values reflect the presence or absence of points in each cell. This grid is processed through a U-Net-like convolutional neural network to produce oriented bounding boxes enclosing the vehicles present in the scene. As with YOLOv2 and YOLOv3, the 2D input grid is subdivided into regularly spaced regions containing one bounding box proposal. This subdivision is finer-grained than that of YOLOv2 and YOLOv3, which leads to a higher number of bounding boxes per vehicle. A post-processing technique called Non-Maximum Suppression (NMS) is thus necessary to remove duplicate detections.

## 3. Proposed Detectors

In this section, we present five vehicle detectors on occupancy grid maps. We first describe the various possible design options in terms of architecture, dimension of the output, labeling strategy and post-processing. The feature extraction parts of the proposed architectures are inspired from existing object detector backbones mentioned in Section 2. A custom convolutional architecture is branched on top of these feature extractors to process features into oriented bounding boxes around vehicles. In comparison, most of the original object detectors used as inspirations produce non-oriented bounding boxes (with the exception of the PIXOR [18] object detector). A detailed depiction of the differences from the original backbones is presented in Section 3.3. At the end of this section, a table summarizes the different options selected for each proposed detector.

### 3.1. Description of the Output

**Figure 1:**
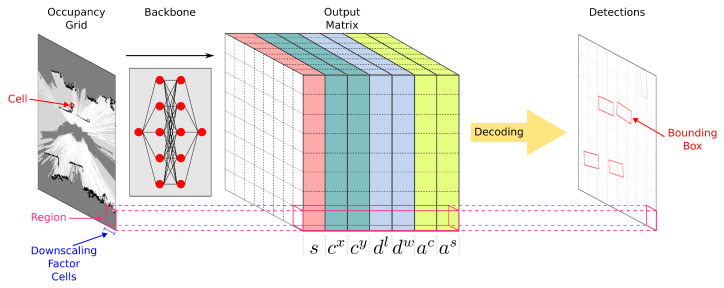
Illustration of the input occupancy grid map, the output matrix encoding the proposed bounding boxes and the detections once decoded from the output matrix.

Figure 1 represents the entire detection pipeline from input occupancy grids to bounding boxes. The backbones of the proposed detectors are fed with an occupancy grid map and produce a matrix representing a regular subdivision of the occupancy grid map into squared regions as an output. Each region encodes one potential bounding box. The size of the output matrix is the size of the input occupancy grid map divided by the downscaling factor of the convolutional neural network. For example, with a downscaling factor of 32, each region corresponds to a square of 32×32 cells of the input occupancy grid map. As each region encodes at most one bounding box, the downscaling factor of the convolutional neural network drives the capacity of the detector to predict near bounding boxes.

Practically, each region contains a tuple $r=(s,c^{x},c^{y},d^{l},d^{w},a^{c},a^{s})$ encoding a potential bounding box:

- s∈[0,1] is a confidence score used to represent the confidence of the detector in the correctness of the predicted bounding box. A sigmoid is used as the last layer activation function to ensure a confidence score in [0,1];
- $c^{x}$ and $c^{y}$ represent the offset of the center (x,y) of the bounding box from the upper left corner of the region;
- $d^{l}$ and $d^{w}$ represent the logarithm of the dimensions (l,w) of the bounding box;
- $a^{c}$ and $a^{s}$ represent the cosine and sine of the heading angle θ of the bounding box.

After the inference, each region contains an encoded bounding box along with a confidence score. However, only a few represent real objects present in the occupancy grid map. The confidence score value allows discrimination between correct and incorrect bounding boxes by keeping only bounding boxes with a confidence score above a user-defined threshold. However, this can result in multiple confident bounding boxes corresponding to the same object in the occupancy grid map, especially when a small downscaling factor is used. A post-processing method known as Non-Maximum Suppression can be used to remove duplicate bounding boxes at the cost of a slightly increased computational overhead.

### 3.2. Training

Training the detectors in a supervised way requires providing the expected output matrix for each input occupancy grid map. In datasets, ground truth bounding boxes for vehicles are presented as a list of labels (x,y,l,w,θ) encoding bounding boxes for each vehicle present in the occupancy grid map. Two strategies for transforming these ground truth bounding boxes to output matrices exist: one-to-one label-to-region mapping and one-to-many label-to-region mapping. One-to-one mapping is the process of finding the output region containing the center of the ground truth bounding box and assigning this region to this label. It is a reasonable choice when the space covered by an output region is about the size of a bounding box. One-to-many mapping consists in assigning all output regions strictly included in the ground truth bounding box to this label. It is useful when the space covered by an output region is a lot smaller than the size of a bounding boxes because many different output regions are good candidates for encoding the bounding box. However, this mapping requires Non-Maximum Suppression during the inference step as the network is trained to let multiple regions predict a bounding box for the same vehicle.

Let us denote the coordinates of the top left corner of the region as $\left({\mathrm{origin}}^{x},{\mathrm{origin}}^{y}\right)$ and the length of the side of the region as side. At the end, each output region assigned to a label (x,y,l,w,θ) is encoded as the following:

$$\begin{array}{cc} s & =1\\ c^{x} & =\frac{\left(x-{\mathrm{origin}}^{x}\right)}{\mathrm{side}}\\ c^{y} & =\frac{\left(y-{\mathrm{origin}}^{y}\right)}{\mathrm{side}}\\ d^{l} & =\log\;l\\ d^{w} & =\log\;w\\ a^{c} & =\cos\theta \\ a^{s} & =\sin\theta \end{array}$$

Output regions not assigned to any label are encoded as (0,0,0,0,0,0,0).

The loss used ℒ is the sum of the classification loss ${ℒ}_{cls}$, which is the binary cross entropy loss on the confidence scores, *s* and the regression loss ${ℒ}_{reg}$, which is the smooth L1 loss over the regression parameters $\left(c^{x},c^{y},d^{l},d^{w},a^{c},a^{s}\right)$, for the regions assigned to a ground truth bounding box. Mathematically speaking, for a predicted output matrix $\hat{y}=\left({\hat{r}}_{i,j}\right)$ and the corresponding ground truth matrix $y=(r_{i,j})$ with $r_{i,j}=\left(s_{i,j},c_{i,j}^{x},c_{i,j}^{y},d_{i,j}^{l},d_{i,j}^{w},a_{i,j}^{c},a_{i,j}^{s}\right)$, the loss is the following:

$$\begin{array}{cc} ℒ(\hat{y},y) & =\sum_{i,j}{ℒ}_{cls}\left({\hat{s}}_{i,j},s_{i,j}\right)+\sum_{i,j}s_{i,j}\left[\sum_{p\in \{c^{x},c^{y},d^{l},d^{w},a^{c},a^{s}\}}{ℒ}_{reg}\left({\hat{p}}_{i,j},p_{i,j}\right)\right]\\ \mathrm{where}\;{ℒ}_{cls}\left(a,b\right) & =-(a\log(b)+(1-a)\log(1-b))\\ {ℒ}_{reg}\left(a,b\right) & =\left\{\begin{array}{cc} 0.5\cdot {\left|a-b\right|}^{2}, & \mathrm{if}\;|a-b|\leq 1\\ |a-b|-0.5, & \mathrm{if}\;|a-b|>1 \end{array}\right. \end{array}$$

All models are trained using Adam [33], a stochastic gradient descent method which is computationally efficient and well suited for training large networks. The learning rate is 0.001 and the exponential decay rate is 0.9 for the first moment estimate and 0.999 for the second moment estimate. The training is stopped after the first epoch which degrades the validation loss, a process known as early stopping.

### 3.3. Backbones

Four different convolutional architectures are used as backbones to process the input occupancy grid maps into output matrices containing bounding boxes. Proposed backbones are inspired from YOLOv2’s, YOLOv3’s and PIXOR’s feature extractors. Detailed depictions of the proposed architectures can be found in Appendix A.

YOLOv2’s Darknet-19 backbone and YOLOv3’s Darknet-53 backbone have been chosen for comparison due to their important architectural differences in terms of layers used for downscaling, usage of residual connections and number of trainable parameters. YOLOv4’s CSPDarknet-53 backbone has not been included in this study due to the similarity of the architecture to that of YOLOv3’s Darknet-53. As CSPDarknet-53 could provide better real-time capabilities due to its Cross Stage Partial connections, we postulate that it would provide only a small improvement over Darknet-53 in terms of Average Precision metrics.

**GRID-YOLOv2**: The first architecture used is inspired by the architecture of YOLOv2 [16]. An occupancy grid map is processed through an alternation of convolutional layers extracting features from the grid and max-pooling layers downsampling the features to the target downscaling factor of 32. Two parallel convolutional layers process the features into confidence scores and regression parameters using a sigmoid activation function for the first layer and a linear activation function for the second layer.

Compared to the original backbone, the number of filters of each convolutional layer is divided by two. This modification divides the number of trainable weights present inside the backbone by four, making it closer to other backbones and thus allowing a fairer comparison. Moreover, the output of the backbone described in Section 3.1 is different from the output of the original detector. First, there is no class prediction per region because only vehicles are detected. Second, bounding boxes predicted are oriented through the prediction of an additional heading angle per bounding box. Finally, as there is no notion of depth on occupancy grids, the objects cannot overlap. As a consequence, it is unnecessary to predict multiple bounding boxes per region.

**GRID-YOLOv2-noMP**: This architecture is designed to have a downscaling factor of 16 while being similar to the previous architecture. This goal is attained through the removal of the last max-pooling layer in GRID-YOLOv2, thus halving the downscaling factor while retaining the same overall architecture with the same number of trainable weights. Since the role of max-pooling layers are to compress the activations of the previous layer into a smaller tensor, removing them allows for a larger output tensor with more regions.

**GRID-YOLOv3**: This architecture, inspired from YOLOv3 [17], is an alternation of blocks of layers extracting features interleaved with convolutional layers with a stride of 2 for downsampling the features to the target downscaling factor of 32. Feature extracting blocks are composed of convolutional layers with skip connections to help the training of the network. This architecture is also similar to YOLOv4’s [25] feature extraction backbone without Cross Stage Partial connections.

The same modifications made to GRID-YOLOv2 apply here as well. The number of filters of each convolutional layers is halved and the output is modified to produce one oriented bounding box per region.

**GRID-PIXOR**: This architecture, taken from PIXOR [18] and used without modifications, computes three levels of granularity of features. The features are further combined as in the U-Net [34] neural network architecture. Features are extracted using residual blocks containing convolutional layers of increasing depth.

### 3.4. Configuration of the Proposed Detectors

Table 1 summarizes the configuration of the five detectors studied: **D1**, **D2**, **D3**, **D4** and **D5**. Their architectures are specified, along with the related number of trainable weights and downscaling factor. The label-to-region mapping method used for constructing the ground truth output matrices from ground truth bounding boxes needed for training the network is specified. Finally, the presence or absence of the NMS post-processing used after inference to remove duplicate detections is also mentioned.

## 4. Experimental Setup

The various experimental choices made to evaluate the performance of the proposed detectors are presented in this section. The methodology used to construct the dataset used for training and evaluating the detectors is described first. The second part presents a description of the different metrics used to evaluate the quality of the detections and the real-time capabilities of the detectors.

### 4.1. Dataset

The supervised training of vehicle detectors on occupancy grid maps requires a dataset containing both sensor-independent occupancy grid maps and ground truth oriented bounding boxes for vehicles. To the best of our knowledge, such a dataset does not exist. However, a dataset containing occupancy grid maps can be constructed from a dataset containing range sensors measurements using the approach proposed in [35].

In this study, the original labeled dataset used is Waymo Open [19] because it contains the highest number of annotated samples along with five different sensors that can be fused into occupancy grid maps. It is composed of 1000 segments, with each segment containing point clouds for 20 s long driving situations. The five LiDARs mounted on the vehicle (top, front, side left, side right and rear) output point clouds at 10 Hz, making each 20 s-long segment a sequence of roughly 200 frames containing 5 point clouds each. Moreover, each frame is labeled with 3D bounding boxes for vehicles, which can be transformed into 2D boxes by orthogonal projection onto the ground plane to eliminate the *z* coordinate and the height of the bounding boxes. The dataset is split into 798 segments (158,081 frames) for training, 101 segments (20,007 frames) for validation and 101 segments (19,980 frames) for testing. The frames inside a segment are temporally contiguous and thus highly correlated. As such, in order to keep the different splits independent of each other, the segments are uniformly distributed inside each split but all frames from the same segment are guaranteed to belong to the same split.

**Figure 2:**
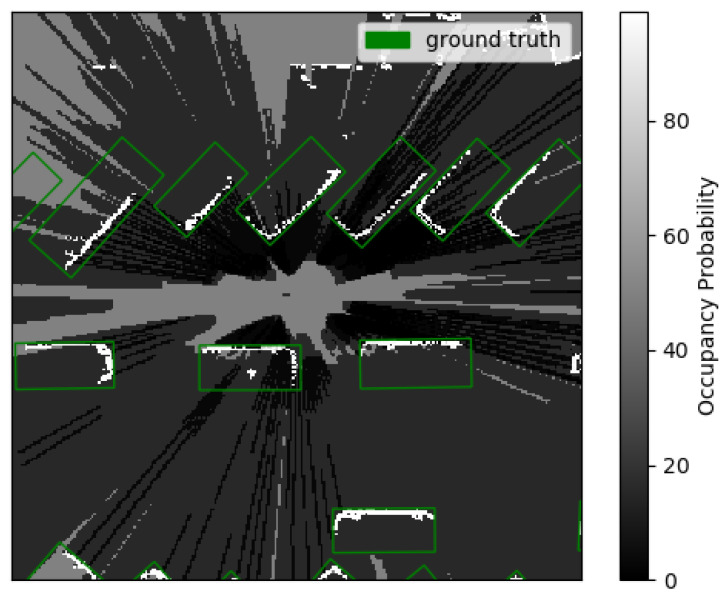
Occupancy grid map produced from a Waymo Open [19] frame. Green bounding boxes are ground truth vehicles. Occupancy probability is encoded in grayscale.

Occupancy grid maps produced from this dataset cover an area of 25.6m×25.6m centered on the ego vehicle with a grid cell size of 0.1m×0.1m. Only points between 0.5 and 0.7 m above ground are considered for the computation of occupancy grid maps; this setting does not retain points reflected by the ground but only points reflected by vehicles. The generated occupancy grid maps are thus 256×256 2D grids with 1 channel for the occupancy probability in [0,1], discretized with a padding of 0.01. Figure 2 presents such an occupancy grid map with ground truth bounding boxes for vehicles.

### 4.2. Metrics

According to the main objective of real-time detection of vehicles on occupancy grid maps, a balance must be achieved between the quality of the detections produced and the latency for processing an occupancy grid map. Two metrics are thus proposed to evaluate the detectors: the Average Precision evaluates the quality of the detections produced; and the inference timings, post-processing timings and the frame rate are used to quantify the real-time capabilities of the proposed detectors.

Average Precision (AP) is the classical metric used to evaluate object detectors. As such, it is used in this study to measure the quality of the bounding boxes predicted by the detectors. This metric has first been used to evaluate the performance of binary classifiers and has been extended later to evaluate object detectors. Average Precision relies on the classification of the bounding boxes produced by the detector into true positives and false positives by comparing them to ground truth bounding boxes. In order to do this classification, output bounding boxes (called detections) must be associated with ground truth bounding boxes by computing the intersection over union (IoU) of these two bounding boxes. If this IoU is greater than a chosen threshold, the detection is associated to the ground truth bounding box and thus considered as a true positive. Otherwise, the detection is considered as a false positive. The choice of the IoU threshold defines the expected geometrical similarity between detections and ground truth bounding boxes. In this study, two Average Precision metrics are computed with two different IoU thresholds: $AP_{0.5}$ with an IoU threshold of 0.5 and $AP_{0.7}$ with an IoU threshold of 0.7. These two variants are both classically used to evaluate object detectors [18,24]. As $AP_{0.7}$ requires a higher IoU between detections and ground truth bounding boxes than $AP_{0.5}$, it is expected that for a given detector, $AP_{0.7}$ is less than $AP_{0.5}$.

Real-time capabilities of the proposed detectors are measured in Frames Per Second (FPS). The frame rate depends on inference time and post-processing time. The timings of all the detectors are measured on the same computer equipped with an Intel Core i7-10850H as the CPU and an Nvidia Quadro RTX 3000 Mobile as the GPU. All code is written in the Julia programming language, which is compiled just-in-time before the code executes. Before timing, inference and post-processing functions are called for one time to trigger compilation before the timing loop starts. Inference is done with the TensorFlow [36] deep learning framework. When measuring the post-processing time, the confidence score threshold chosen to consider detections as positive is important as it changes the number of detections that need to be compared against each other in the Non-Maximum Suppression algorithm. This confidence score threshold has been chosen to be 0.5 in this study.

## 5. Results

This section presents the results obtained with the five proposed detectors. A quantitative evaluation provides the metrics obtained on the test split of the dataset. Following this evaluation is a discussion of the reasons behind these metrics values using Precision–Recall curves and illustrations of detections on different occupancy grid maps.

### 5.1. Quantitative Results

Table 2 presents the quality metrics ${\mathrm{AP}}_{0.5}$ and ${\mathrm{AP}}_{0.7}$ and the real-time metrics measured for the five proposed detectors.

Regarding the Average Precision scores, the detectors can be classified into two groups. **D2**, **D3** and **D5** offer the best quality detections with an ${\mathrm{AP}}_{0.7}$ in the interval of [0.77,0.82], whereas **D1** and **D4** produce much worse detections in the interval of [0.50,0.55].

Regarding the total detection time on a single occupancy grid map, two different behaviors are observed in the first group:

- **D5** has an inference time of 45 ms and a post-processing time of 5 ms.
- **D2** and **D3**, which are based on a different convolutional architecture, have an inference time of 11 ms and a negligible post-processing time in the case of **D3** (and no post-processing at all for **D2**).

In the other group:

- **D1** has the same inference time of 11 ms as **D2** and **D3**.
- **D4** has an inference time of 35 ms.

Singularly, **D3**, which differs from **D2** only by the use of a NMS post-processing after the inference, loses 0.02 in ${\mathrm{AP}}_{0.7}$ compared to **D2**.

These metrics point towards the designation of **D5** as the detector providing the best detections and **D2** (respectively, **D3**) as the detector providing the best balance between detections quality and frame rate if duplicate detections are allowed (respectively, not allowed). A qualitative analysis required to better understand these metrics is presented in the following section.

### 5.2. Analysis of the Quality of the Detections

The Average Precision, computed as the mean of the Precision, depends on the Recall; a closer look at the Precision–Recall curves helps in understanding the obtained metrics.

**Figure 3:**
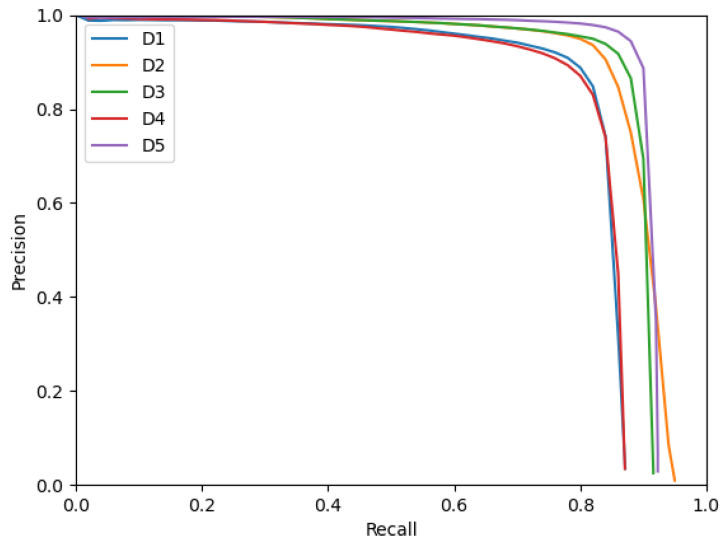
Precision–Recall curves for detectors with an IoU threshold of 0.5.

Figure 3 presents the Precision–Recall curves of the five detectors obtained when considering that a proposed bounding box is a true positive if it has an IoU with a ground truth bounding box greater or equal to 0.5. The worse performing detectors, **D1** and **D4**, can be observed as the two almost coincidental lower curves. On the other hand, **D2**, **D3** and **D5** present different behaviors. **D5** is able to achieve a slightly greater Precision than **D3** on the same Recall range. **D2** performs worse than **D3** and **D5** on the Recall range of [0,0.9] but is able to reach a greater maximal Recall of 0.95. **D3** and **D5** both use a NMS duplicate deletion algorithm, whereas **D2** is the same as **D3**, with the exception of not having NMS post-processing. One could hypothesize that the higher attainable Recall of **D2** comes from the presence of correct detections (true positives) in some occupancy grid maps mislabeled by the NMS algorithm as duplicate detections.

**Figure 4:**
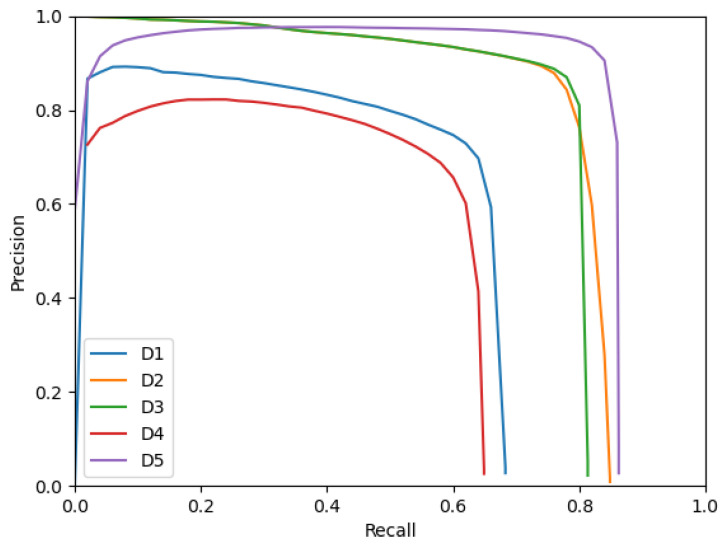
Precision–Recall curves for detectors with an IoU threshold of 0.7.

Figure 4 presents the Precision–Recall curves of the detectors with an IoU threshold used for association between detections and ground truth bounding boxes of 0.7. Similar behaviors to the ones noted for Figure 3 can be observed here as well. Additionally, the curves of **D1**, **D4** and **D5** decrease when approaching a Recall of 0. This behavior is unusual for object detectors because lowering the Recall is done by raising the confidence score threshold used to select the bounding boxes produced by the detectors as positives. Therefore, raising the confidence score threshold should lead to more confident bounding boxes selected and a lower number of false positives (and thus a higher Precision). In this case, the behavior denotes that **D1**, **D4** and **D5** sometimes produce incorrect bounding boxes (false positives) with a confidence score close to 1. This behavior makes **D5** less performative than **D2** and **D3** in the high confidence score threshold regime and thus explains why **D5** performs very similarly to **D2** and **D3**; this is true despite having a better Precision than **D2** and **D3** in the low confidence score threshold regime.

**Figure 5:**
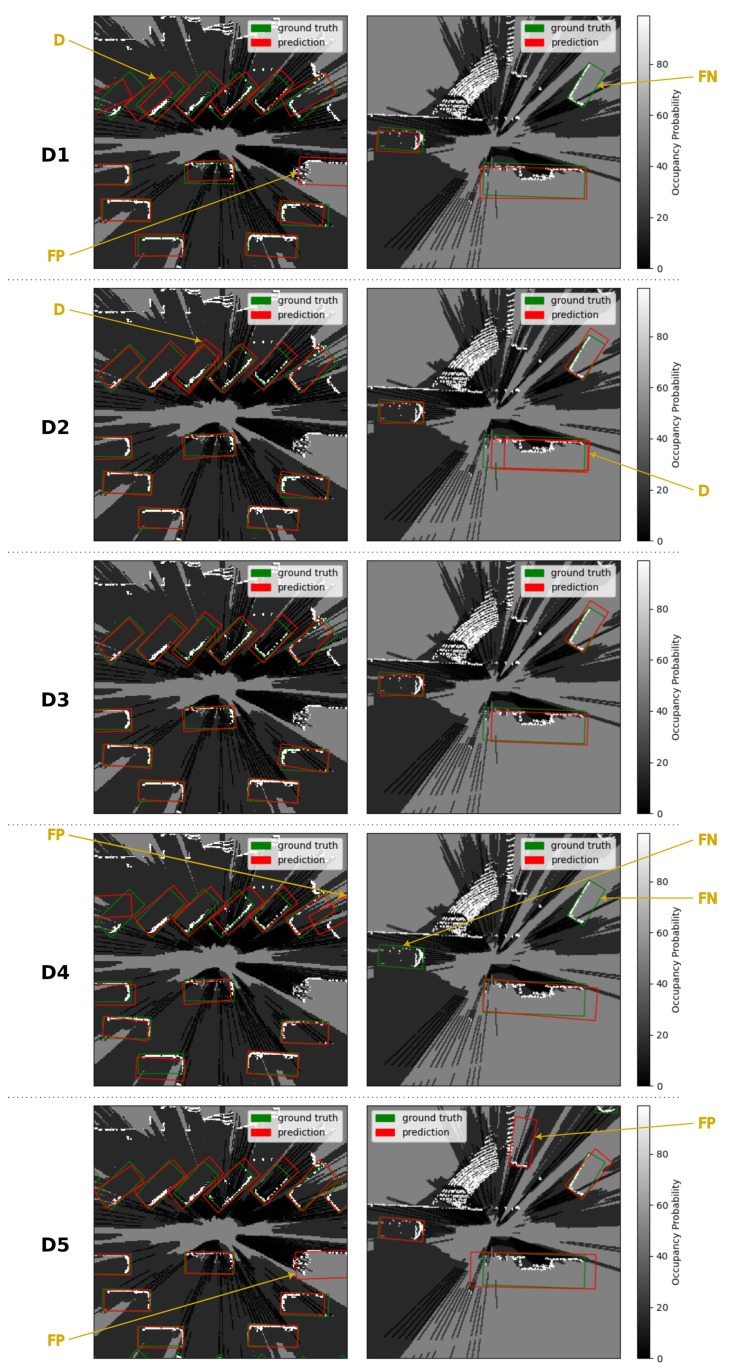
Examples of predictions. Occupancy probability is encoded in grayscale. False positive (FP), false negative (FN) and duplicate (D) detections are highlighted with golden arrows.

Figure 5 presents detections produced by the five detectors on two different occupancy grid maps. The first occupancy grid map corresponds to a dense traffic situation with a lot of vehicles close to each other. The second occupancy grid map corresponds to a turn at an intersection with two cars and one bus. These examples illustrate the behaviors seen on the Precision–Recall curves. Highly confident false positives produced by **D1**, **D4** and **D5** appear in both occupancy grid maps. False negatives in the occupancy grid map in the right column illustrate the difficulties of **D1** and **D4** to detect all vehicles in the environment. **D2** produces duplicate detections in both occupancy grid maps. These detections are correctly eliminated by the Non-Maximum Suppression algorithm of **D3**.

### 5.3. Analysis of the Timings

The timings obtained on the different detectors vary greatly between 11 ms and 50 ms. These timings and the associated frame rates are of utmost importance for real-time operation and are discussed in the following section. The five detectors presented can be divided into three different groups:

- **D1**, **D2** and **D3** have the same total detection time of 11 ms. **D1** uses GRID-YOLOv2 as the convolutional architecture and **D2** and **D3** use the same convolutional architecture of GRID-YOLOv2-noMP. This only differs from the former in the deletion of the last max-pooling layer;
- **D4**, which relies on GRID-YOLOv3 as the convolutional architecture, has a total detection time of 35 ms;
- Finally, **D5** has a total detection time of 50 ms and is based on the GRID-PIXOR convolutional architecture.

Two steps account for the total detection time: the inference and the post-processing steps. The inference time seems to follow the complexity of the convolutional architectures presented in Appendix A. First, GRID-YOLOv2 and GRID-YOLOv2-noMP have a simple feedforward architecture without skip connections, which allows for a fast inference time. On the other hand, GRID-YOLOv3 contains skip connections and GRID-PIXOR even uses different levels of details for the feature maps computed. Thus, **D4** and **D5** have higher inference times than those of **D1**, **D2** and **D3**. Remarkably, **D5** contains fewer trainable weights than **D1**, **D2** and **D3**, but is nonetheless slower due to the more sequential nature of GRID-PIXOR.

The post-processing time is the time required to delete duplicate detections using the Non-Maximum Suppression method when used in the detector. The NMS algorithm compares each predicted bounding box with the others and is thus quadratic in the number of predicted bounding boxes. The number of predicted bounding boxes for one detector itself depends on the size of the output matrix of the neural network. The size of the output matrix is itself quadratic in the inverse of the downscaling factor, as explained in Section 3.1. The cost of the NMS algorithm is thus proportional to the inverse of the downscaling factor raised to the power four. As an example, **D5** has a downscaling factor that is four times smaller than that of **D3**; thus, its post-processing takes approximately 256 times longer. Therefore, the post-processing time, which is negligible in the case of **D3**, accounts for 10% of the total detection time of **D5**.

These timings are further used to compute the frame rate of each detector measured in Frames Per Second (FPS). A higher frame rate is more compatible with real-time operation as the latency of the perception system is reduced. Furthermore, a detector with a higher frame rate leaves more room to a low-power implementation than a detector with a lower frame rate. **D1**, **D2** and **D3** are especially fast regarding this metric with a frame rate of 91 FPS, compared to, respectively, 29 FPS and 20 FPS for **D4** and **D5**.

## 6. Conclusions

This article proposes five models for vehicle detection on occupancy grid maps inspired by related work in object detection on matrix format inputs. All of these detectors output oriented bounding boxes for vehicles detected on occupancy grid maps with a focus on the balance between the Average Precision of the detections produced and the frame rate achieved by the detectors. One detector (**D5**) provides the best Average Precision metrics at the cost of a low frame rate of 20 FPS on a desktop GPU. On the other hand, two detectors (**D2** and **D3**) are able to provide comparable detections to those of the best detector **D5** for a higher frame rate. One of these two detectors is equipped with a post-processing method that deletes duplicate detections for negligible overhead.

In a realistic setup, those detectors should be able to provide detections in real-time on a low-power hardware. The frame rate of the two proposed detectors **D2** and **D3** is expected to meet real-time requirements, even with the lower frame rate due to the limited computing power of an embedded hardware.

The proposed detectors focus on the detection of vehicles on occupancy grid maps. A logical next research direction is the extension of these detectors to other classes of moving objects such as pedestrians or bicycles. Due to the small and blurred shape of such objects on occupancy grid maps, we postulate that detecting such objects requires the usage of more information. In particular, we plan to study the integration of temporal information into the detectors by using sequences containing present and past occupancy grid maps as input.

## Figures and Tables

**Table 1:** Summary of the five detectors studied.

Detector	Downscaling Factor	Convolutional Architecture	Trainable Weights	Label-to-Region Mapping	Post-Processing
D1	32	GRID-YOLOv2	4,961,463	One-to-one	N/A
D2	16	GRID-YOLOv2-noMP	4,961,463	One-to-one	N/A
D3	16	GRID-YOLOv2-noMP	4,961,463	One-to-one	NMS
D4	32	GRID-YOLOv3	10,158,679	One-to-one	N/A
D5	4	GRID-PIXOR	2,133,379	One-to-many	NMS

**Table 2:** Average Precisions, inference, post-processing times and Frame Rate. The GPU used for measuring inference times is an Nvidia Quadro RTX 3000 Mobile. Post-processing times are computed with a chosen confidence score threshold of 0.5 as explained in Section 4.2. Bold numbers indicate best results in each column.

Detector	${\mathrm{AP}}_{0.7}$	${\mathrm{AP}}_{0.5}$	Inference Time	Post-Processing Time	Total Detection Time	Frame Rate
D1	0.55	0.82	**11 ms**	**N/A**	**11 ms**	**91 FPS**
D2	0.79	0.89	**11 ms**	**N/A**	**11 ms**	**91 FPS**
D3	0.77	0.89	**11 ms**	**≈0 ms**	**≈11 ms**	**91 FPS**
D4	0.50	0.82	35 ms	**N/A**	35ms	29 FPS
D5	**0.82**	**0.90**	45 ms	5 ms	50ms	20 FPS

**Figure A1:**
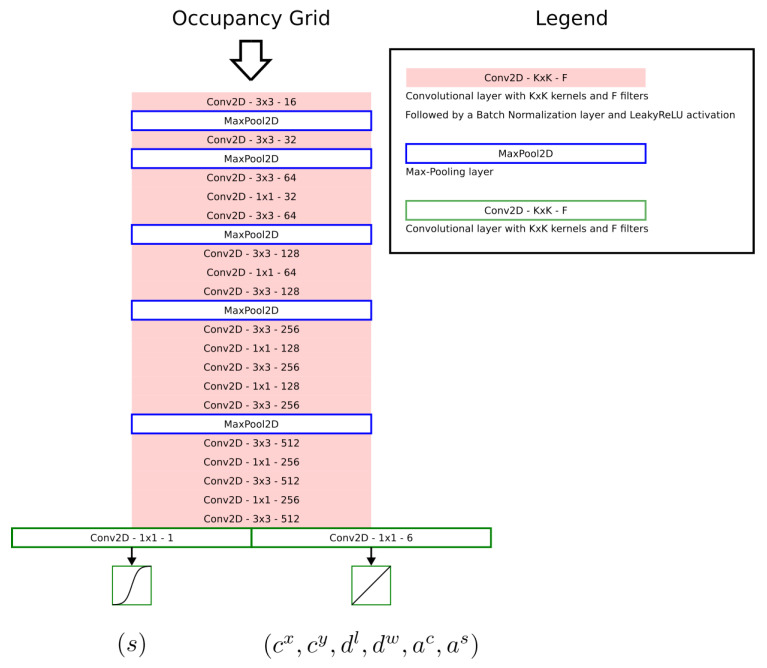
Architecture of GRID-YOLOv2 containing 4,961,463 trainable parameters.

**Figure A2:**
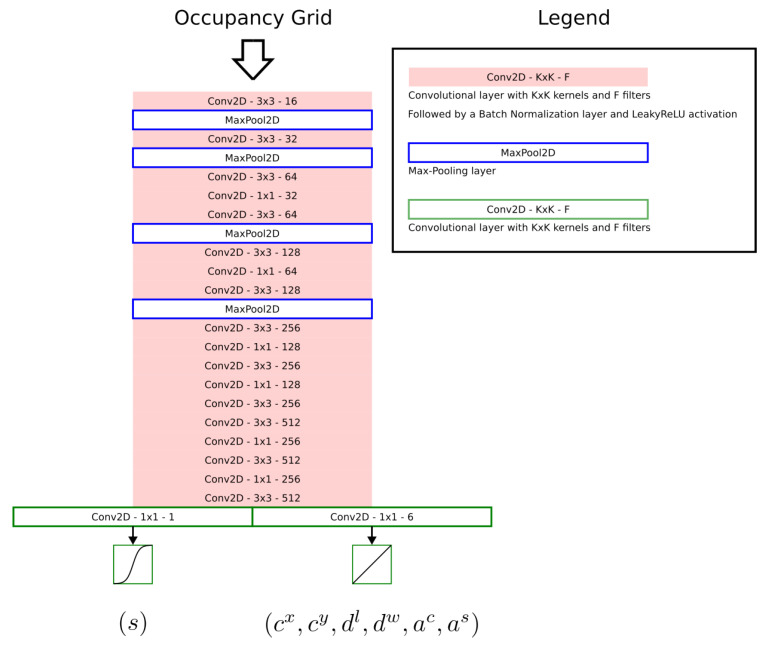
Architecture of GRID-YOLOv2-noMP containing 4,961,463 trainable parameters.

**Figure A3:**
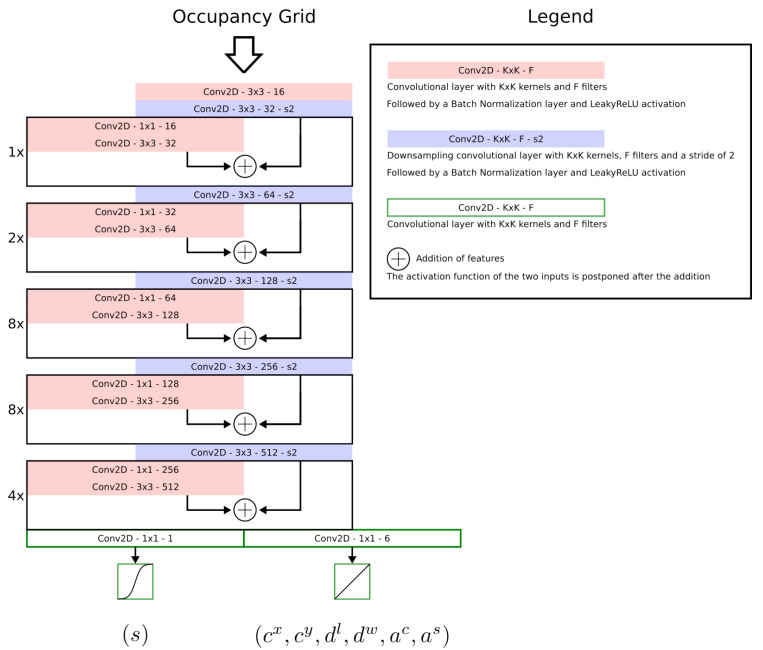
Architecture of GRID-YOLOv3 containing 10,158,679 trainable parameters.

**Figure A4:**
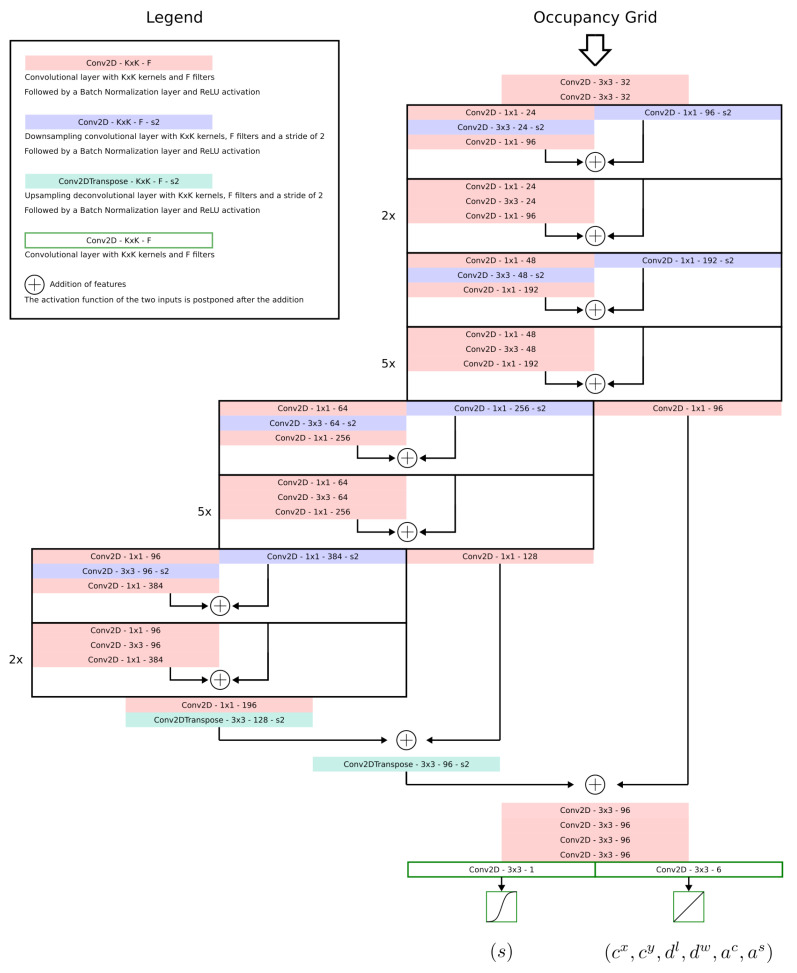
Architecture of GRID-PIXOR containing 2,132,091 trainable parameters.

## Data Availability

Publicly available datasets were analyzed in this study. This data can be found here: waymo.com/open (accessed on 25 January 2021).

## Abbreviations

The following abbreviations are used in this manuscript:

LiDAR	Light Detection And Ranging
NMS	Non-Maximum Suppression
AP	Average Precision
FPS	Frames Per Second
IoU	Intersection over Union
FP	False Positive detection
FN	False Negative detection
D	Duplicate detection
CPU	Central Processing Unit
GPU	Graphics Processing Unit
SAR	Synthetic Aperture Radar
CSP	Cross Stage Partial
